# 1-Benzyl-3-[(dimethyl­amino)methyl­ene]-4-phenyl-1*H*-1,5-benzodiazepine-2(3*H*)-thione

**DOI:** 10.1107/S160053681001175X

**Published:** 2010-04-02

**Authors:** Daouda Ballo, Rachid Bouhfid, Hafid Zouihri, El Mokhtar Essassi, Seik Weng Ng

**Affiliations:** a Laboratoire de Chimie Organique Hétérocyclique, Pôle de Compétences Pharmacochimie, Université Mohammed V-Agdal, BP 1014 Avenue Ibn Batout, Rabat, Morocco; b CNRST Division UATRS, Angle Allal Fassi/FAR, BP 8027 Hay Riad, 10000 Rabat, Morocco; c Department of Chemistry, University of Malaya, 50603 Kuala Lumpur, Malaysia

## Abstract

The title compound, C_25_H_23_N_3_S, crystallizes with two independent mol­ecules in the asymmetric unit. The seven-membered fused-ring adopts a boat conformation, with the two bridgehead C atoms representing the stern and the C atom bearing the exocyclic double bond the prow.

## Related literature

For background to the synthesis and biological activity of pyrazolo[4,3-*c*]triazolo[4,3-*a*][1,5] benzodiazepines, see: Ahab­chane *et al.* (2001 ▶).
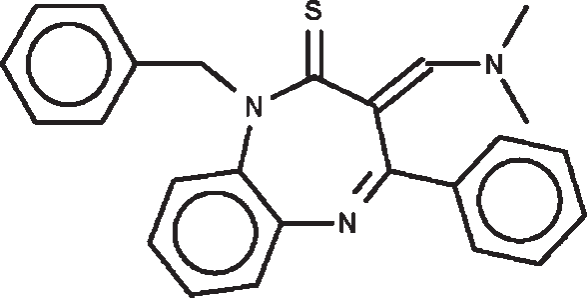


## Experimental

### 

#### Crystal data

C_25_H_23_N_3_S
*M* *_r_* = 397.52
Monoclinic, 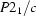

*a* = 26.4740 (8) Å
*b* = 10.2366 (2) Å
*c* = 16.5650 (5) Å
β = 106.136 (1)°
*V* = 4312.3 (2) Å^3^
*Z* = 8
Mo *K*α radiation
μ = 0.17 mm^−1^
*T* = 293 K
0.38 × 0.35 × 0.28 mm

#### Data collection

Bruker X8 APEX2 diffractometer
Absorption correction: multi-scan (*SADABS*; Sheldrick, 1996 ▶) *T*_min_ = 0.940, *T*_max_ = 0.955
34616 measured reflections
7429 independent reflections
5019 reflections with *I* > 2σ(*I*)
*R*_int_ = 0.047

#### Refinement

*R*[*F* ^2^ > 2σ(*F* ^2^)] = 0.045
*wR*(*F* ^2^) = 0.150
*S* = 1.03
7429 reflections
527 parameters
H-atom parameters constrained
Δρ_max_ = 0.19 e Å^−3^
Δρ_min_ = −0.28 e Å^−3^

### 

Data collection: *APEX2* (Bruker, 2008 ▶); cell refinement: *SAINT* (Bruker, 2008 ▶); data reduction: *SAINT*; program(s) used to solve structure: *SHELXS97* (Sheldrick, 2008 ▶); program(s) used to refine structure: *SHELXL97* (Sheldrick, 2008 ▶); molecular graphics: *X-SEED* (Barbour, 2001 ▶); software used to prepare material for publication: *publCIF* (Westrip, 2010 ▶).

## Supplementary Material

Crystal structure: contains datablocks global, I. DOI: 10.1107/S160053681001175X/bt5234sup1.cif

Structure factors: contains datablocks I. DOI: 10.1107/S160053681001175X/bt5234Isup2.hkl

Additional supplementary materials:  crystallographic information; 3D view; checkCIF report

## Figures and Tables

**Fig. 1.**
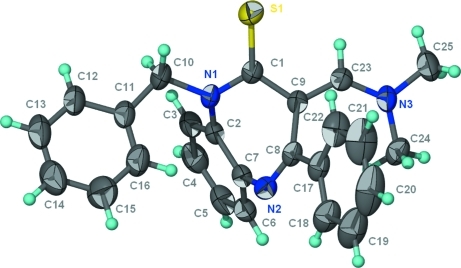
Anisotropic displacement ellipsoid plot (Barbour, 2001) of one independent molecule of C25H23N3S at the 50% probability level; hydrogen atoms are drawn as arbitrary radius.

**Fig. 2.**
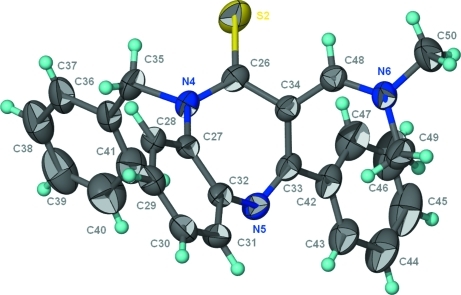
Anisotropic displacement ellipsoid plot (Barbour, 2001) of the second independent molecule of C25H23N3S at the 50% probability level; hydrogen atoms are drawn as arbitrary radius.

## supplementary crystallographic information

### Comment

The background to the class of precusor compounds is explained in a report on the syntheses and biological properties of pyrazolo[4,3-*c*]triazolo[4,3-*a*][1,5] benzodiazepines (Ahabchane *et al.*, 2001). The methylene linkage of the seven-membered fused ring of 1-benzyl-4-phenyl-1,5-benzodiazepine-2-thione is exceptionally reactive; in the present study, this portion reacts with *N,N*-dimethylformamide–dimethylacetal to furnish a double bond.

The seven-membered fused-ring in C_25_H_23_N_3_S (Scheme I, Figs. 1 & 2) adopts a boat conformation (with the two phenylene carbons representing the stern and the carbon atom bearing the exocyclic double-bond the prow) in the two independent molecules. The fused-ring features exocyclic double (C═C and C═S) bonds on adjacent carbon occupants but the C(═C)–C(═S) fragment is twisted [-34.8 (3), 40.1 (3) °].

### Experimental

1-Benzyl-4-phenyl-1,5-benzodiazepine-2-thione (1 g) and an excess of *N,N*-dimethylformamide dimethylacetal (DMF–DMA, 2.5 ml) were heated for 12 hours. The solid that formed upon cooling the mixture was recrystallized from ethanol to afford colorless crystals in 65% yield.

### Refinement

H-atoms were placed in calculated positions (C—H 0.93–0.97 Å) and were included in the refinement in the riding model approximation, with *U*(H) set to 1.2*U*(C).

### Crystal data

**Table:**

C_25_H_23_N_3_S	*F*(000) = 1680
*M**_r_* = 397.52	*D*_x_ = 1.225 Mg m^−^^3^
Monoclinic, *P*2_1_/*c*	Mo *K*α radiation, λ = 0.71073 Å
Hall symbol: -P 2ybc	Cell parameters from 7420 reflections
*a* = 26.4740 (8) Å	θ = 2.6–21.9°
*b* = 10.2366 (2) Å	µ = 0.17 mm^−^^1^
*c* = 16.5650 (5) Å	*T* = 293 K
β = 106.136 (1)°	Block, colorless
*V* = 4312.3 (2) Å^3^	0.38 × 0.35 × 0.28 mm
*Z* = 8	

### Data collection

**Table:**

Bruker X8 APEX2 diffractometer	7429 independent reflections
Radiation source: fine-focus sealed tube	5019 reflections with *I* > 2σ(*I*)
graphite	*R*_int_ = 0.047
φ and ω scans	θ_max_ = 24.8°, θ_min_ = 0.8°
Absorption correction: multi-scan (*SADABS*; Sheldrick, 1996)	*h* = −31→31
*T*_min_ = 0.940, *T*_max_ = 0.955	*k* = −8→12
34616 measured reflections	*l* = −19→19

### Refinement

**Table:**

Refinement on *F*^2^	Primary atom site location: structure-invariant direct methods
Least-squares matrix: full	Secondary atom site location: difference Fourier map
*R*[*F*^2^ > 2σ(*F*^2^)] = 0.045	Hydrogen site location: inferred from neighbouring sites
*wR*(*F*^2^) = 0.150	H-atom parameters constrained
*S* = 1.03	*w* = 1/[σ^2^(*F*_o_^2^) + (0.0771*P*)^2^ + 0.7415*P*] where *P* = (*F*_o_^2^ + 2*F*_c_^2^)/3
7429 reflections	(Δ/σ)_max_ = 0.001
527 parameters	Δρ_max_ = 0.19 e Å^−^^3^
0 restraints	Δρ_min_ = −0.28 e Å^−^^3^

### Special details

**Table:**

Geometry. All esds (except the esd in the dihedral angle between two l.s. planes) are estimated using the full covariance matrix. The cell esds are taken into account individually in the estimation of esds in distances, angles and torsion angles; correlations between esds in cell parameters are only used when they are defined by crystal symmetry. An approximate (isotropic) treatment of cell esds is used for estimating esds involving l.s. planes.

### Fractional atomic coordinates and isotropic or equivalent isotropic displacement parameters (Å^2^)

**Table:**

	*x*	*y*	*z*	*U*_iso_*/*U*_eq_	
S1	0.68147 (3)	0.23703 (6)	0.88424 (4)	0.0681 (2)	
S2	0.82290 (3)	0.28053 (7)	0.73502 (5)	0.0811 (3)	
N1	0.66628 (8)	0.46928 (18)	0.94582 (11)	0.0537 (5)	
N2	0.60213 (7)	0.65564 (19)	0.83124 (12)	0.0503 (5)	
N3	0.66494 (8)	0.48619 (18)	0.65939 (11)	0.0507 (5)	
N4	0.81709 (7)	0.52681 (18)	0.78290 (12)	0.0505 (5)	
N5	0.89168 (7)	0.6994 (2)	0.73201 (12)	0.0553 (5)	
N6	0.84302 (8)	0.48869 (18)	0.51100 (12)	0.0540 (5)	
C1	0.66570 (9)	0.3953 (2)	0.87702 (14)	0.0457 (5)	
C2	0.68024 (9)	0.6051 (2)	0.94640 (14)	0.0474 (6)	
C3	0.72558 (11)	0.6494 (3)	1.00447 (15)	0.0646 (7)	
H3	0.7460	0.5914	1.0433	0.078*	
C4	0.74049 (11)	0.7774 (3)	1.00508 (18)	0.0726 (8)	
H4	0.7710	0.8059	1.0442	0.087*	
C5	0.71064 (12)	0.8640 (3)	0.94813 (18)	0.0670 (7)	
H5	0.7214	0.9504	0.9474	0.080*	
C6	0.66488 (10)	0.8226 (2)	0.89239 (15)	0.0563 (6)	
H6	0.6439	0.8825	0.8558	0.068*	
C7	0.64933 (9)	0.6922 (2)	0.88965 (14)	0.0460 (5)	
C8	0.60280 (8)	0.5527 (2)	0.78745 (14)	0.0457 (5)	
C9	0.64898 (8)	0.4652 (2)	0.79744 (13)	0.0423 (5)	
C10	0.66833 (13)	0.4077 (3)	1.02656 (16)	0.0730 (8)	
H10A	0.7037	0.4168	1.0636	0.088*	
H10B	0.6614	0.3150	1.0173	0.088*	
C11	0.63036 (11)	0.4626 (2)	1.06983 (15)	0.0565 (6)	
C12	0.63693 (12)	0.4266 (3)	1.15333 (15)	0.0678 (8)	
H12	0.6657	0.3759	1.1809	0.081*	
C13	0.60148 (15)	0.4651 (3)	1.1952 (2)	0.0844 (10)	
H13	0.6058	0.4386	1.2504	0.101*	
C14	0.55973 (16)	0.5424 (3)	1.1560 (2)	0.0943 (11)	
H14	0.5359	0.5691	1.1846	0.113*	
C15	0.55316 (14)	0.5804 (3)	1.0742 (2)	0.0948 (10)	
H15	0.5250	0.6338	1.0476	0.114*	
C16	0.58831 (12)	0.5394 (3)	1.03122 (18)	0.0753 (8)	
H16	0.5833	0.5643	0.9756	0.090*	
C17	0.55336 (9)	0.5137 (3)	0.72353 (15)	0.0566 (6)	
C18	0.51377 (10)	0.6058 (3)	0.69462 (18)	0.0767 (8)	
H18	0.5179	0.6903	0.7161	0.092*	
C19	0.46822 (13)	0.5715 (5)	0.6338 (2)	0.1040 (12)	
H19	0.4418	0.6331	0.6144	0.125*	
C20	0.46198 (15)	0.4468 (6)	0.6023 (2)	0.1212 (17)	
H20	0.4315	0.4245	0.5609	0.145*	
C21	0.50005 (15)	0.3556 (4)	0.6313 (2)	0.1088 (13)	
H21	0.4952	0.2707	0.6106	0.131*	
C22	0.54606 (11)	0.3887 (3)	0.69144 (18)	0.0759 (8)	
H22	0.5722	0.3263	0.7103	0.091*	
C23	0.67079 (9)	0.4318 (2)	0.73465 (13)	0.0453 (5)	
H23	0.6932	0.3600	0.7461	0.054*	
C24	0.63639 (10)	0.6057 (3)	0.63075 (16)	0.0633 (7)	
H24A	0.6359	0.6587	0.6783	0.095*	
H24B	0.6010	0.5850	0.5996	0.095*	
H24C	0.6533	0.6526	0.5953	0.095*	
C25	0.69212 (14)	0.4298 (3)	0.60257 (19)	0.0846 (10)	
H25A	0.7099	0.3514	0.6269	0.127*	
H25B	0.7173	0.4913	0.5933	0.127*	
H25C	0.6671	0.4093	0.5499	0.127*	
C26	0.82869 (9)	0.4423 (2)	0.72705 (15)	0.0511 (6)	
C27	0.80627 (9)	0.6622 (2)	0.76243 (13)	0.0459 (5)	
C28	0.75899 (9)	0.7160 (2)	0.76785 (15)	0.0537 (6)	
H28	0.7347	0.6633	0.7837	0.064*	
C29	0.74769 (10)	0.8452 (3)	0.75021 (15)	0.0603 (7)	
H29	0.7157	0.8792	0.7532	0.072*	
C30	0.78350 (11)	0.9250 (2)	0.72817 (15)	0.0593 (7)	
H30	0.7759	1.0129	0.7166	0.071*	
C31	0.83043 (10)	0.8741 (2)	0.72333 (15)	0.0571 (6)	
H31	0.8547	0.9289	0.7093	0.068*	
C32	0.84279 (9)	0.7420 (2)	0.73898 (14)	0.0488 (6)	
C33	0.89356 (9)	0.5921 (2)	0.69224 (14)	0.0492 (6)	
C34	0.84874 (9)	0.5015 (2)	0.66108 (14)	0.0459 (5)	
C35	0.80751 (10)	0.4803 (3)	0.86157 (15)	0.0629 (7)	
H35A	0.7961	0.3900	0.8542	0.076*	
H35B	0.7790	0.5310	0.8721	0.076*	
C36	0.85436 (11)	0.4894 (2)	0.93730 (15)	0.0566 (6)	
C37	0.85080 (14)	0.4299 (3)	1.01068 (17)	0.0754 (8)	
H37	0.8201	0.3861	1.0111	0.091*	
C38	0.89202 (18)	0.4346 (4)	1.0831 (2)	0.0983 (11)	
H38	0.8894	0.3918	1.1312	0.118*	
C39	0.93670 (18)	0.5021 (4)	1.0842 (2)	0.1075 (13)	
H39	0.9642	0.5062	1.1333	0.129*	
C40	0.94095 (15)	0.5642 (4)	1.0124 (2)	0.1018 (11)	
H40	0.9712	0.6108	1.0131	0.122*	
C41	0.89962 (12)	0.5567 (3)	0.93863 (18)	0.0741 (8)	
H41	0.9027	0.5975	0.8901	0.089*	
C42	0.94507 (10)	0.5544 (3)	0.68004 (15)	0.0612 (7)	
C43	0.98279 (10)	0.6497 (3)	0.68304 (18)	0.0791 (9)	
H43	0.9760	0.7359	0.6944	0.095*	
C44	1.03046 (13)	0.6171 (5)	0.6692 (2)	0.1057 (13)	
H44	1.0557	0.6813	0.6718	0.127*	
C45	1.04039 (14)	0.4910 (6)	0.6519 (2)	0.1153 (15)	
H45	1.0722	0.4698	0.6417	0.138*	
C46	1.00412 (15)	0.3958 (4)	0.6495 (2)	0.1076 (13)	
H46	1.0114	0.3099	0.6385	0.129*	
C47	0.95619 (12)	0.4269 (3)	0.6635 (2)	0.0807 (9)	
H47	0.9315	0.3617	0.6618	0.097*	
C48	0.83331 (9)	0.4494 (2)	0.58218 (15)	0.0497 (6)	
H48	0.8128	0.3746	0.5771	0.060*	
C49	0.86997 (11)	0.6091 (3)	0.50126 (17)	0.0675 (7)	
H49A	0.8668	0.6709	0.5432	0.101*	
H49B	0.8544	0.6448	0.4464	0.101*	
H49C	0.9065	0.5910	0.5078	0.101*	
C50	0.82724 (13)	0.4069 (3)	0.43621 (17)	0.0795 (9)	
H50A	0.8016	0.3445	0.4426	0.119*	
H50B	0.8574	0.3617	0.4288	0.119*	
H50C	0.8124	0.4606	0.3879	0.119*	

### Atomic displacement parameters (Å^2^)

**Table:**

	*U*^11^	*U*^22^	*U*^33^	*U*^12^	*U*^13^	*U*^23^
S1	0.0990 (6)	0.0484 (4)	0.0570 (4)	0.0109 (3)	0.0218 (4)	0.0068 (3)
S2	0.1156 (7)	0.0479 (4)	0.0950 (6)	0.0057 (4)	0.0544 (5)	0.0176 (4)
N1	0.0777 (14)	0.0499 (12)	0.0360 (10)	0.0084 (10)	0.0200 (10)	0.0073 (9)
N2	0.0505 (12)	0.0540 (12)	0.0469 (11)	0.0068 (9)	0.0140 (9)	0.0049 (10)
N3	0.0650 (13)	0.0514 (12)	0.0384 (11)	−0.0074 (10)	0.0190 (9)	−0.0014 (9)
N4	0.0601 (12)	0.0521 (12)	0.0428 (11)	−0.0041 (9)	0.0201 (9)	0.0067 (9)
N5	0.0469 (12)	0.0668 (14)	0.0522 (12)	−0.0125 (10)	0.0137 (9)	−0.0001 (10)
N6	0.0661 (13)	0.0537 (12)	0.0442 (11)	−0.0009 (10)	0.0188 (10)	0.0026 (9)
C1	0.0470 (13)	0.0487 (14)	0.0415 (13)	−0.0003 (10)	0.0124 (10)	0.0045 (10)
C2	0.0578 (14)	0.0514 (14)	0.0355 (12)	0.0063 (11)	0.0170 (11)	−0.0035 (10)
C3	0.0728 (18)	0.0748 (19)	0.0413 (14)	0.0154 (14)	0.0078 (13)	−0.0114 (13)
C4	0.0693 (18)	0.084 (2)	0.0619 (18)	−0.0082 (16)	0.0130 (15)	−0.0288 (16)
C5	0.0786 (19)	0.0661 (18)	0.0637 (17)	−0.0133 (15)	0.0322 (16)	−0.0191 (15)
C6	0.0731 (17)	0.0503 (15)	0.0528 (15)	0.0058 (13)	0.0299 (14)	−0.0002 (12)
C7	0.0508 (14)	0.0508 (14)	0.0406 (12)	0.0040 (11)	0.0197 (11)	−0.0010 (10)
C8	0.0440 (13)	0.0563 (15)	0.0373 (12)	−0.0018 (10)	0.0119 (10)	0.0091 (11)
C9	0.0462 (13)	0.0430 (12)	0.0369 (12)	−0.0046 (10)	0.0103 (10)	0.0000 (9)
C10	0.111 (2)	0.0674 (17)	0.0441 (15)	0.0195 (16)	0.0280 (15)	0.0180 (13)
C11	0.0801 (18)	0.0498 (14)	0.0432 (14)	−0.0087 (13)	0.0230 (13)	0.0008 (11)
C12	0.104 (2)	0.0582 (16)	0.0448 (15)	−0.0079 (14)	0.0270 (15)	−0.0002 (12)
C13	0.135 (3)	0.073 (2)	0.0582 (18)	−0.016 (2)	0.047 (2)	−0.0024 (15)
C14	0.126 (3)	0.092 (2)	0.090 (3)	−0.006 (2)	0.071 (2)	0.002 (2)
C15	0.093 (2)	0.109 (3)	0.096 (3)	0.0123 (19)	0.049 (2)	0.023 (2)
C16	0.083 (2)	0.094 (2)	0.0553 (17)	0.0005 (17)	0.0297 (16)	0.0145 (15)
C17	0.0455 (14)	0.0812 (19)	0.0427 (13)	−0.0084 (13)	0.0117 (11)	0.0102 (13)
C18	0.0481 (16)	0.115 (2)	0.0646 (18)	0.0049 (16)	0.0111 (14)	0.0197 (16)
C19	0.053 (2)	0.179 (4)	0.073 (2)	−0.001 (2)	0.0064 (17)	0.027 (3)
C20	0.064 (2)	0.223 (5)	0.065 (2)	−0.050 (3)	−0.0010 (19)	0.010 (3)
C21	0.086 (3)	0.152 (4)	0.080 (2)	−0.062 (3)	0.009 (2)	−0.017 (2)
C22	0.0687 (19)	0.090 (2)	0.0650 (18)	−0.0293 (16)	0.0116 (15)	−0.0051 (16)
C23	0.0532 (14)	0.0384 (12)	0.0424 (13)	−0.0068 (10)	0.0104 (11)	−0.0029 (10)
C24	0.0687 (17)	0.0696 (17)	0.0533 (15)	0.0031 (13)	0.0199 (13)	0.0191 (13)
C25	0.138 (3)	0.0695 (19)	0.0611 (18)	0.0058 (18)	0.0527 (19)	−0.0032 (14)
C26	0.0494 (14)	0.0507 (14)	0.0538 (14)	0.0007 (11)	0.0153 (11)	0.0104 (11)
C27	0.0538 (14)	0.0475 (14)	0.0360 (12)	−0.0107 (11)	0.0116 (10)	−0.0044 (10)
C28	0.0549 (15)	0.0589 (16)	0.0514 (14)	−0.0091 (12)	0.0215 (12)	−0.0048 (12)
C29	0.0670 (17)	0.0583 (17)	0.0571 (16)	0.0022 (13)	0.0200 (13)	−0.0092 (13)
C30	0.0779 (19)	0.0493 (14)	0.0479 (14)	−0.0014 (13)	0.0130 (13)	−0.0060 (11)
C31	0.0679 (17)	0.0529 (16)	0.0482 (14)	−0.0193 (13)	0.0126 (12)	−0.0032 (11)
C32	0.0516 (14)	0.0545 (15)	0.0387 (12)	−0.0125 (11)	0.0099 (11)	−0.0041 (10)
C33	0.0459 (14)	0.0597 (16)	0.0422 (13)	−0.0026 (11)	0.0123 (11)	0.0111 (11)
C34	0.0485 (13)	0.0441 (13)	0.0472 (13)	0.0018 (10)	0.0165 (11)	0.0057 (10)
C35	0.0753 (18)	0.0688 (17)	0.0516 (15)	−0.0050 (13)	0.0290 (14)	0.0135 (12)
C36	0.0785 (18)	0.0496 (14)	0.0451 (14)	0.0119 (13)	0.0225 (13)	0.0030 (11)
C37	0.118 (3)	0.0643 (17)	0.0503 (17)	0.0153 (16)	0.0335 (17)	0.0036 (13)
C38	0.149 (4)	0.092 (3)	0.0510 (19)	0.028 (2)	0.023 (2)	0.0039 (17)
C39	0.130 (3)	0.121 (3)	0.054 (2)	0.034 (3)	−0.004 (2)	−0.002 (2)
C40	0.090 (2)	0.121 (3)	0.082 (3)	0.007 (2)	0.002 (2)	−0.002 (2)
C41	0.074 (2)	0.089 (2)	0.0551 (17)	0.0070 (16)	0.0124 (15)	0.0074 (15)
C42	0.0475 (15)	0.089 (2)	0.0473 (14)	0.0042 (14)	0.0142 (12)	0.0129 (13)
C43	0.0509 (16)	0.121 (3)	0.0668 (18)	−0.0120 (16)	0.0186 (14)	0.0005 (17)
C44	0.054 (2)	0.182 (4)	0.084 (2)	−0.012 (2)	0.0234 (18)	0.011 (3)
C45	0.057 (2)	0.205 (5)	0.089 (3)	0.034 (3)	0.0286 (19)	0.028 (3)
C46	0.086 (3)	0.142 (3)	0.104 (3)	0.053 (2)	0.042 (2)	0.038 (2)
C47	0.0653 (19)	0.092 (2)	0.088 (2)	0.0247 (16)	0.0270 (17)	0.0269 (18)
C48	0.0523 (14)	0.0441 (13)	0.0548 (15)	0.0024 (10)	0.0185 (12)	0.0046 (11)
C49	0.0755 (18)	0.0737 (18)	0.0563 (16)	−0.0098 (14)	0.0232 (14)	0.0123 (13)
C50	0.109 (2)	0.0770 (19)	0.0531 (17)	−0.0052 (17)	0.0239 (16)	−0.0049 (14)

### Geometric parameters (Å, °)

**Table:**

S1—C1	1.669 (2)	C21—H21	0.9300
S2—C26	1.672 (2)	C22—H22	0.9300
N1—C1	1.365 (3)	C23—H23	0.9300
N1—C2	1.438 (3)	C24—H24A	0.9600
N1—C10	1.466 (3)	C24—H24B	0.9600
N2—C8	1.283 (3)	C24—H24C	0.9600
N2—C7	1.402 (3)	C25—H25A	0.9600
N3—C23	1.334 (3)	C25—H25B	0.9600
N3—C24	1.447 (3)	C25—H25C	0.9600
N3—C25	1.454 (3)	C26—C34	1.472 (3)
N4—C26	1.362 (3)	C27—C28	1.393 (3)
N4—C27	1.437 (3)	C27—C32	1.400 (3)
N4—C35	1.474 (3)	C28—C29	1.370 (3)
N5—C33	1.289 (3)	C28—H28	0.9300
N5—C32	1.400 (3)	C29—C30	1.375 (3)
N6—C48	1.336 (3)	C29—H29	0.9300
N6—C49	1.455 (3)	C30—C31	1.370 (4)
N6—C50	1.457 (3)	C30—H30	0.9300
C1—C9	1.457 (3)	C31—C32	1.399 (3)
C2—C7	1.386 (3)	C31—H31	0.9300
C2—C3	1.389 (3)	C33—C42	1.484 (3)
C3—C4	1.368 (4)	C33—C34	1.481 (3)
C3—H3	0.9300	C34—C48	1.365 (3)
C4—C5	1.374 (4)	C35—C36	1.502 (3)
C4—H4	0.9300	C35—H35A	0.9700
C5—C6	1.370 (4)	C35—H35B	0.9700
C5—H5	0.9300	C36—C41	1.377 (4)
C6—C7	1.394 (3)	C36—C37	1.386 (3)
C6—H6	0.9300	C37—C38	1.380 (5)
C8—C9	1.487 (3)	C37—H37	0.9300
C8—C17	1.491 (3)	C38—C39	1.365 (5)
C9—C23	1.366 (3)	C38—H38	0.9300
C10—C11	1.497 (4)	C39—C40	1.381 (5)
C10—H10A	0.9700	C39—H39	0.9300
C10—H10B	0.9700	C40—C41	1.397 (4)
C11—C16	1.368 (4)	C40—H40	0.9300
C11—C12	1.395 (3)	C41—H41	0.9300
C12—C13	1.370 (4)	C42—C47	1.382 (4)
C12—H12	0.9300	C42—C43	1.387 (4)
C13—C14	1.367 (5)	C43—C44	1.384 (4)
C13—H13	0.9300	C43—H43	0.9300
C14—C15	1.374 (5)	C44—C45	1.364 (6)
C14—H14	0.9300	C44—H44	0.9300
C15—C16	1.384 (4)	C45—C46	1.361 (6)
C15—H15	0.9300	C45—H45	0.9300
C16—H16	0.9300	C46—C47	1.388 (4)
C17—C22	1.378 (4)	C46—H46	0.9300
C17—C18	1.392 (4)	C47—H47	0.9300
C18—C19	1.384 (4)	C48—H48	0.9300
C18—H18	0.9300	C49—H49A	0.9600
C19—C20	1.371 (6)	C49—H49B	0.9600
C19—H19	0.9300	C49—H49C	0.9600
C20—C21	1.360 (6)	C50—H50A	0.9600
C20—H20	0.9300	C50—H50B	0.9600
C21—C22	1.384 (4)	C50—H50C	0.9600
			
C1—N1—C2	119.02 (18)	H24B—C24—H24C	109.5
C1—N1—C10	120.81 (19)	N3—C25—H25A	109.5
C2—N1—C10	117.75 (19)	N3—C25—H25B	109.5
C8—N2—C7	117.40 (19)	H25A—C25—H25B	109.5
C23—N3—C24	125.0 (2)	N3—C25—H25C	109.5
C23—N3—C25	119.3 (2)	H25A—C25—H25C	109.5
C24—N3—C25	115.5 (2)	H25B—C25—H25C	109.5
C26—N4—C27	121.32 (18)	N4—C26—C34	116.01 (19)
C26—N4—C35	121.48 (19)	N4—C26—S2	122.16 (17)
C27—N4—C35	116.62 (19)	C34—C26—S2	121.78 (18)
C33—N5—C32	118.36 (19)	C28—C27—C32	119.3 (2)
C48—N6—C49	125.0 (2)	C28—C27—N4	119.3 (2)
C48—N6—C50	119.8 (2)	C32—C27—N4	121.3 (2)
C49—N6—C50	115.2 (2)	C29—C28—C27	121.0 (2)
N1—C1—C9	114.40 (19)	C29—C28—H28	119.5
N1—C1—S1	122.29 (17)	C27—C28—H28	119.5
C9—C1—S1	123.29 (17)	C28—C29—C30	120.3 (2)
C7—C2—C3	119.6 (2)	C28—C29—H29	119.9
C7—C2—N1	121.0 (2)	C30—C29—H29	119.9
C3—C2—N1	119.4 (2)	C31—C30—C29	119.5 (2)
C4—C3—C2	120.6 (3)	C31—C30—H30	120.2
C4—C3—H3	119.7	C29—C30—H30	120.2
C2—C3—H3	119.7	C30—C31—C32	121.7 (2)
C3—C4—C5	120.2 (3)	C30—C31—H31	119.1
C3—C4—H4	119.9	C32—C31—H31	119.1
C5—C4—H4	119.9	N5—C32—C31	117.3 (2)
C6—C5—C4	119.7 (3)	N5—C32—C27	124.5 (2)
C6—C5—H5	120.2	C31—C32—C27	118.1 (2)
C4—C5—H5	120.2	N5—C33—C42	117.3 (2)
C5—C6—C7	121.1 (2)	N5—C33—C34	124.8 (2)
C5—C6—H6	119.5	C42—C33—C34	117.9 (2)
C7—C6—H6	119.5	C48—C34—C26	118.4 (2)
C2—C7—C6	118.7 (2)	C48—C34—C33	125.2 (2)
C2—C7—N2	122.8 (2)	C26—C34—C33	114.7 (2)
C6—C7—N2	118.5 (2)	N4—C35—C36	114.4 (2)
N2—C8—C9	124.9 (2)	N4—C35—H35A	108.7
N2—C8—C17	118.3 (2)	C36—C35—H35A	108.7
C9—C8—C17	116.8 (2)	N4—C35—H35B	108.7
C23—C9—C1	118.7 (2)	C36—C35—H35B	108.7
C23—C9—C8	125.1 (2)	H35A—C35—H35B	107.6
C1—C9—C8	115.48 (18)	C41—C36—C37	118.5 (3)
N1—C10—C11	114.6 (2)	C41—C36—C35	123.8 (2)
N1—C10—H10A	108.6	C37—C36—C35	117.6 (3)
C11—C10—H10A	108.6	C38—C37—C36	121.1 (3)
N1—C10—H10B	108.6	C38—C37—H37	119.4
C11—C10—H10B	108.6	C36—C37—H37	119.4
H10A—C10—H10B	107.6	C39—C38—C37	120.2 (3)
C16—C11—C12	118.4 (3)	C39—C38—H38	119.9
C16—C11—C10	124.0 (2)	C37—C38—H38	119.9
C12—C11—C10	117.4 (2)	C38—C39—C40	119.9 (4)
C13—C12—C11	120.8 (3)	C38—C39—H39	120.0
C13—C12—H12	119.6	C40—C39—H39	120.0
C11—C12—H12	119.6	C39—C40—C41	119.8 (4)
C14—C13—C12	120.2 (3)	C39—C40—H40	120.1
C14—C13—H13	119.9	C41—C40—H40	120.1
C12—C13—H13	119.9	C36—C41—C40	120.5 (3)
C13—C14—C15	119.7 (3)	C36—C41—H41	119.7
C13—C14—H14	120.1	C40—C41—H41	119.7
C15—C14—H14	120.1	C47—C42—C43	118.7 (3)
C14—C15—C16	120.1 (3)	C47—C42—C33	121.8 (2)
C14—C15—H15	119.9	C43—C42—C33	119.5 (3)
C16—C15—H15	119.9	C44—C43—C42	120.3 (3)
C11—C16—C15	120.7 (3)	C44—C43—H43	119.8
C11—C16—H16	119.7	C42—C43—H43	119.8
C15—C16—H16	119.7	C45—C44—C43	120.0 (4)
C22—C17—C18	119.0 (3)	C45—C44—H44	120.0
C22—C17—C8	121.6 (2)	C43—C44—H44	120.0
C18—C17—C8	119.4 (3)	C46—C45—C44	120.5 (4)
C19—C18—C17	119.9 (3)	C46—C45—H45	119.7
C19—C18—H18	120.0	C44—C45—H45	119.7
C17—C18—H18	120.0	C45—C46—C47	120.1 (4)
C20—C19—C18	120.1 (4)	C45—C46—H46	120.0
C20—C19—H19	120.0	C47—C46—H46	120.0
C18—C19—H19	120.0	C42—C47—C46	120.3 (3)
C21—C20—C19	120.4 (4)	C42—C47—H47	119.9
C21—C20—H20	119.8	C46—C47—H47	119.9
C19—C20—H20	119.8	N6—C48—C34	130.4 (2)
C20—C21—C22	120.2 (4)	N6—C48—H48	114.8
C20—C21—H21	119.9	C34—C48—H48	114.8
C22—C21—H21	119.9	N6—C49—H49A	109.5
C17—C22—C21	120.4 (3)	N6—C49—H49B	109.5
C17—C22—H22	119.8	H49A—C49—H49B	109.5
C21—C22—H22	119.8	N6—C49—H49C	109.5
N3—C23—C9	130.0 (2)	H49A—C49—H49C	109.5
N3—C23—H23	115.0	H49B—C49—H49C	109.5
C9—C23—H23	115.0	N6—C50—H50A	109.5
N3—C24—H24A	109.5	N6—C50—H50B	109.5
N3—C24—H24B	109.5	H50A—C50—H50B	109.5
H24A—C24—H24B	109.5	N6—C50—H50C	109.5
N3—C24—H24C	109.5	H50A—C50—H50C	109.5
H24A—C24—H24C	109.5	H50B—C50—H50C	109.5
			
C2—N1—C1—C9	−34.4 (3)	C27—N4—C26—C34	22.1 (3)
C10—N1—C1—C9	163.6 (2)	C35—N4—C26—C34	−166.9 (2)
C2—N1—C1—S1	147.05 (18)	C27—N4—C26—S2	−160.33 (17)
C10—N1—C1—S1	−14.9 (3)	C35—N4—C26—S2	10.6 (3)
C1—N1—C2—C7	64.1 (3)	C26—N4—C27—C28	124.0 (2)
C10—N1—C2—C7	−133.4 (2)	C35—N4—C27—C28	−47.4 (3)
C1—N1—C2—C3	−115.5 (2)	C26—N4—C27—C32	−57.5 (3)
C10—N1—C2—C3	47.0 (3)	C35—N4—C27—C32	131.1 (2)
C7—C2—C3—C4	−1.3 (4)	C32—C27—C28—C29	0.3 (3)
N1—C2—C3—C4	178.4 (2)	N4—C27—C28—C29	178.9 (2)
C2—C3—C4—C5	0.1 (4)	C27—C28—C29—C30	−1.2 (4)
C3—C4—C5—C6	2.1 (4)	C28—C29—C30—C31	0.5 (4)
C4—C5—C6—C7	−3.2 (4)	C29—C30—C31—C32	1.0 (4)
C3—C2—C7—C6	0.2 (3)	C33—N5—C32—C31	−134.9 (2)
N1—C2—C7—C6	−179.43 (19)	C33—N5—C32—C27	46.8 (3)
C3—C2—C7—N2	−177.7 (2)	C30—C31—C32—N5	179.7 (2)
N1—C2—C7—N2	2.7 (3)	C30—C31—C32—C27	−1.8 (3)
C5—C6—C7—C2	2.0 (3)	C28—C27—C32—N5	179.5 (2)
C5—C6—C7—N2	−180.0 (2)	N4—C27—C32—N5	1.0 (3)
C8—N2—C7—C2	−48.7 (3)	C28—C27—C32—C31	1.1 (3)
C8—N2—C7—C6	133.4 (2)	N4—C27—C32—C31	−177.4 (2)
C7—N2—C8—C9	3.7 (3)	C32—N5—C33—C42	175.4 (2)
C7—N2—C8—C17	−178.59 (19)	C32—N5—C33—C34	−7.2 (3)
N1—C1—C9—C23	146.7 (2)	N4—C26—C34—C48	−142.3 (2)
S1—C1—C9—C23	−34.8 (3)	S2—C26—C34—C48	40.1 (3)
N1—C1—C9—C8	−43.1 (3)	N4—C26—C34—C33	51.5 (3)
S1—C1—C9—C8	135.43 (19)	S2—C26—C34—C33	−126.0 (2)
N2—C8—C9—C23	−124.8 (2)	N5—C33—C34—C48	132.1 (3)
C17—C8—C9—C23	57.4 (3)	C42—C33—C34—C48	−50.5 (3)
N2—C8—C9—C1	65.6 (3)	N5—C33—C34—C26	−62.9 (3)
C17—C8—C9—C1	−112.1 (2)	C42—C33—C34—C26	114.5 (2)
C1—N1—C10—C11	−133.9 (2)	C26—N4—C35—C36	97.5 (3)
C2—N1—C10—C11	63.9 (3)	C27—N4—C35—C36	−91.2 (3)
N1—C10—C11—C16	15.8 (4)	N4—C35—C36—C41	11.7 (4)
N1—C10—C11—C12	−168.0 (2)	N4—C35—C36—C37	−170.6 (2)
C16—C11—C12—C13	1.3 (4)	C41—C36—C37—C38	−1.7 (4)
C10—C11—C12—C13	−175.1 (3)	C35—C36—C37—C38	−179.5 (3)
C11—C12—C13—C14	−1.6 (5)	C36—C37—C38—C39	2.1 (5)
C12—C13—C14—C15	0.6 (5)	C37—C38—C39—C40	−0.9 (6)
C13—C14—C15—C16	0.8 (6)	C38—C39—C40—C41	−0.5 (6)
C12—C11—C16—C15	0.1 (4)	C37—C36—C41—C40	0.2 (4)
C10—C11—C16—C15	176.3 (3)	C35—C36—C41—C40	177.9 (3)
C14—C15—C16—C11	−1.1 (5)	C39—C40—C41—C36	0.9 (5)
N2—C8—C17—C22	−161.3 (2)	N5—C33—C42—C47	159.0 (2)
C9—C8—C17—C22	16.6 (3)	C34—C33—C42—C47	−18.6 (3)
N2—C8—C17—C18	19.5 (3)	N5—C33—C42—C43	−22.7 (3)
C9—C8—C17—C18	−162.6 (2)	C34—C33—C42—C43	159.7 (2)
C22—C17—C18—C19	−0.7 (4)	C47—C42—C43—C44	0.3 (4)
C8—C17—C18—C19	178.5 (2)	C33—C42—C43—C44	−178.0 (3)
C17—C18—C19—C20	0.2 (5)	C42—C43—C44—C45	0.5 (5)
C18—C19—C20—C21	0.9 (6)	C43—C44—C45—C46	−1.2 (6)
C19—C20—C21—C22	−1.6 (6)	C44—C45—C46—C47	1.0 (6)
C18—C17—C22—C21	0.1 (4)	C43—C42—C47—C46	−0.6 (4)
C8—C17—C22—C21	−179.1 (3)	C33—C42—C47—C46	177.8 (3)
C20—C21—C22—C17	1.1 (5)	C45—C46—C47—C42	−0.1 (5)
C24—N3—C23—C9	6.3 (4)	C49—N6—C48—C34	−6.0 (4)
C25—N3—C23—C9	−178.8 (2)	C50—N6—C48—C34	172.6 (3)
C1—C9—C23—N3	−174.8 (2)	C26—C34—C48—N6	175.5 (2)
C8—C9—C23—N3	15.9 (4)	C33—C34—C48—N6	−20.0 (4)

## References

[bb1] Ahabchane, N. H., Ibrahimi, S., Salem, M., Essassi, E. M., Amzazi, S. & Benjouad, A. (2001). *C. R. Acad. Sci. Paris Chim* **4**, 917–924.

[bb2] Barbour, L. J. (2001). *J. Supramol. Chem.* **1**, 189–191.

[bb3] Bruker (2008). *APEX2* and *SAINT* Bruker AXS Inc., Madison, Wisconsin, USA.

[bb4] Sheldrick, G. M. (1996). *SADABS* University of Göttingen, Germany.

[bb5] Sheldrick, G. M. (2008). *Acta Cryst.* A**64**, 112–122. 10.1107/S0108767307043930 18156677

[bb6] Westrip, S. P. (2010). *publCIF* In preparation.

